# Supplementary material for: Universal correction of enzymatic sequence bias reveals molecular signatures of protein/DNA interactions
Source: Nucleic Acids Res. 2017 Nov 8;46(2):e9. doi: 10.1093/nar/gkx1053 (PMC5778497; doi:10.1093/nar/gkx1053)
Supplement: Supplementary Data [file gkx1053_supp.zip › nar-00439-met-k-2017-File009.docx]

**Figure S1.** **Graphical representation of k-mer mask optimization via enzyme cut-site model.** We model the enzyme cut bias as a PSWM, of length K and parameterized by $\theta$, shared among across all N possible k-mers. Here $X_{i, j}$ represents the observed j-th base of the i-th k-mer sequence. Each k-mer has an unknown orientation, represented by the random variable $Z_{i}$ and parameterized by $\gamma_{i}$. Furthermore, each k-mer is observed $n_{i}$ times in the data. Thus, the full likelihood of the model is:

$$\mathcal{L(}\gamma, \theta| X,Z) =P(X,Z | \gamma, \theta)= \prod_{i} {P(X_{i}, Z_{i} | \gamma_{i}, \theta)}^{n_{i}}=\prod_{i} \left( P(Z_{i}|\gamma)\prod_{j=1}^{K} P(X_{i, j}| Z_{i}, \theta) \right)^{n_{i}}$$

**Figure S2.** **DNase k-mer mask optimization.** (A) The PSWM resulting from the expectation maximization k-mer optimization method shows that the Information Content of the positions gradually decreases with increasing distance from the DNase nick site. (B) The plot of cumulative Information Content of the positions begins to level off at the tetramer. (C) We plot the decrease in the summed standard deviations across a set of PSWMs as we successively increase the k-mer size relative to the centered DNase nick site.

**Figure S3. Corrected DNase-seq data reveals footprints at a MAX binding site.** (A) The precise location of MAX binding (transparent pink) is inferred from the presence of a MAX recognition motif within a MAX ChIP-seq peak. This binding site shows sharp peaks within the binding site in uncorrected DNase-seq profiles; a footprint is present only in the corrected data. (B) A zoom in of the panel (A) reveals that the composite MAX trace from Figure 4A (shown here as a blue trace) is observed at the MAX binding sites.

**Figure S4.** **Corrected DNase-seq data reveals footprints at a GATA3 binding site.** (A) A 10x zoom in of Figure 2B. (B) The single nucleotide resolution profile reveals that the composite GATA3 trace from Figure 4B (shown here as a blue trace) is observed at the GATA3 binding sites. The molecular signature upstream of GATA3 binding in Figure 4B is observed and enhanced at this binding site at position chr12:64625486.

**Figure S5. *SeqOutBias* corrects Cyanase endonuclease bias.** Each composite profile illustrates the average cut frequency at each position between nucleotides. The blue trace is the raw data and the black trace is the 8-mer corrected data.

**Figure S6. *SeqOutBias* corrects Benzonase endonuclease bias.** The composite profiles for FOXA2, CTCF, and CEBP-beta binding sites illustrate the average cut frequency at each position between nucleotides. The blue trace is the raw data and the black trace is the 8-mer corrected data.

**Figure S7. *SeqOutBias* corrects MNase sequence bias.** The composite profiles for MAX, GATA3, and ELF1 indicate that sequence correction abrogates the sharp peaks in the traces. The blue trace is the raw data and the black trace is the 8-mer corrected data.

**Figure S8. ATAC-seq k-mer mask optimization.** We started with a k-mer mask of 12 X bases flanking each side of the Tn5 insertion site and we systematically changed each X position into a masked N. We plot the decrease in the summed standard deviations across a set of PSWMs for the top 11 positions that contribute to Tn5 sequence bias.

**Figure S9. Tn5 insertion bias is corrected in a ATAC-seq experiment from GM12878 cells.** The composite profiles for SP1, EBF1, and REST indicate that sequence correction dampens the sharp peaks in the traces.

**Figure S10. Tn5 insertion bias is corrected in a ATAC-seq experiment from naked DNA.** We generated ATAC-seq data with naked DNA and we find that the composite profiles for TFs exhibit dampened sharp peaks in the corrected traces.

**Figure S11. Sequence bias drives a sharp peak of PRO-seq signal upstream of exons.** The sharp peak at position -3 found in the CAG splice acceptor profile indicates that cytosine is preferentially incorporated during the nuclear run-on or preferentially ligated during the library preparation.

**Figure S12. *SeqOutBias* corrects PRO-seq sequence bias.** Corrected PRO-seq profiles abrogate the sharp peak at position -3 at CAG consensus exons and the modest difference in intensity in the 5´end of the exon indicates that RNA Polymerase may proceed faster at TAG exons relative to CAG.

**Figure S13. Reverse palindromic hexamers do not exhibit enzymatic DNA end repair and ligation bias.** The DNA end substrates are identical in reverse palindromic nick sites, accounting for the absence of bias.

**Figure S14. DNA sequence drives DNA end repair and ligation preference.** Motif analysis of the 5% most enzymatic DNA end repair and ligation biased 6-mers indicates that an Adenine in position 4 of the 6-mer is preferentially sequenced compared to the oppositely oriented nucleotide in position 3**.**

**Figure S15. Enzymatic nick biases are correlated between DNase-seq experiments and correlated between Cyanase and Benzonase digestion experiments.** These scatter plots show that the enzymatic nick biases, as measured by the *seqOutBias* scale factor, are correlated between DNase experiments and correlated between Cyanase and Benzonase.

**Figure S16. Post-nick enzymatic processing biases of DNase are correlated between experiments and the post nick biases of Cyanase and Benzonase are similar.** The relative bias of all 3-mers sequenced (the ratio of x-axis 3-mer to y-axis 3-mer) for four separate experiments.
